# Association between serum carotenoids and risk of endometriosis evidence from NHANES data

**DOI:** 10.1097/MD.0000000000045470

**Published:** 2025-11-07

**Authors:** Zhuoxia Chen, Tingting Zhang, Tiantian Jin

**Affiliations:** a Department of Gynaecology, Shaoxing Second Hospital, Shaoxing, Zhejiang, China.

**Keywords:** carotenoids, endometriosis, NHANES, oxidative stress, serum biomarkers

## Abstract

Endometriosis is a prevalent gynecological disorder, with its pathogenesis potentially linked to oxidative stress. Carotenoids, as natural antioxidants, may play a role in its prevention. This study aimed to investigate the association between serum carotenoid levels and the prevalence of endometriosis. This cross-sectional analysis utilized data from the National Health and Nutrition Examination Survey 2001 to 2006, including 1825 female participants. Survey-weighted logistic regression models were used to assess the associations between serum levels of α-carotene, β-carotene, lycopene, β-cryptoxanthin, lutein/zeaxanthin, and total carotenoids with endometriosis prevalence, adjusting for age, body mass index, poverty income ratio, education level, race/ethnicity, smoking status, age at menarche, and number of pregnancies. Higher serum levels of α-carotene (odds ratio [OR] = 0.94, 95% confidence interval [CI]: 0.89–0.99, *P* = .048), β-carotene (OR = 0.98, 95% CI: 0.96–1.00, *P* = .038), lycopene (OR = 0.95, 95% CI: 0.91–0.99, *P* = .021), and total carotenoids (OR = 0.99, 95% CI: 0.98–1.00, *P* = .044) were significantly associated with a lower prevalence of endometriosis. However, β-cryptoxanthin (OR = 0.94, 95% CI: 0.88–1.01, *P* = .12) and lutein/zeaxanthin (OR = 1.00, 95% CI: 0.97–1.03, *P* = .92) were not significantly associated with endometriosis. Subgroup analysis revealed a significant interaction between β-carotene levels and age at menarche (*P* for interaction < .05). These findings suggest that higher serum levels of α-carotene, β-carotene, lycopene, and total carotenoids may be associated with a lower prevalence of endometriosis. This study highlights the potential role of carotenoid-rich diets in mitigating endometriosis risk; however, further prospective studies are warranted to confirm these associations and explore underlying biological mechanisms.

## 1. Introduction

Endometriosis is a prevalent gynecological disorder characterized by the ectopic implantation of endometrial-like tissue outside the uterine cavity, most commonly affecting the ovaries, fallopian tubes, and pelvic peritoneum. The global prevalence of endometriosis is estimated to range from 6% to 10% in reproductive-aged women, with rates rising to 50% among women experiencing infertility.^[1,2]^ This condition is a leading cause of chronic pelvic pain and is associated with significant impairment in reproductive health and overall quality of life.^[3,4]^ Although the exact etiology of endometriosis remains unclear, it is thought to result from a complex interplay of retrograde menstruation, immune dysfunction, genetic predisposition, and oxidative stress.^[5–7]^

Oxidative stress has been implicated as a key factor in the pathogenesis of endometriosis, contributing to chronic inflammation, immune dysregulation, and cellular damage. Elevated levels of reactive oxygen species (ROS) in the peritoneal fluid of affected individuals have been associated with increased expression of pro-inflammatory cytokines, including interleukin-6 and tumor necrosis factor-alpha, which may exacerbate disease progression.^[8,9]^ Given the role of oxidative stress in endometriosis, dietary and endogenous antioxidants have been proposed as potential protective factors.

Carotenoids, a diverse group of naturally occurring pigments found in fruits and vegetables, exhibit potent antioxidant and anti-inflammatory properties. Major dietary carotenoids, including α-carotene, β-carotene, β-cryptoxanthin, lutein/zeaxanthin, and lycopene, have been shown to modulate oxidative stress and inflammatory pathways involved in various chronic diseases.^[10]^ While carotenoids have been extensively studied in relation to cardiovascular diseases, metabolic disorders, and certain cancers, their potential role in endometriosis remains largely unexplored. Epidemiological evidence on the association between serum carotenoid levels and endometriosis is currently limited, with inconsistent findings across studies.^[11]^

This study aims to investigate the association between serum carotenoid levels and the prevalence of endometriosis using data from the National Health and Nutrition Examination Survey (NHANES) 2001 to 2006. Additionally, this study examines whether this association is modified by demographic and lifestyle factors, including body mass index (BMI), smoking status, and age at menarche, to provide further epidemiological insights into the potential role of carotenoids in endometriosis prevention.

## 2. Materials and methods

### 2.1. Study design and population

**Figure 1.**
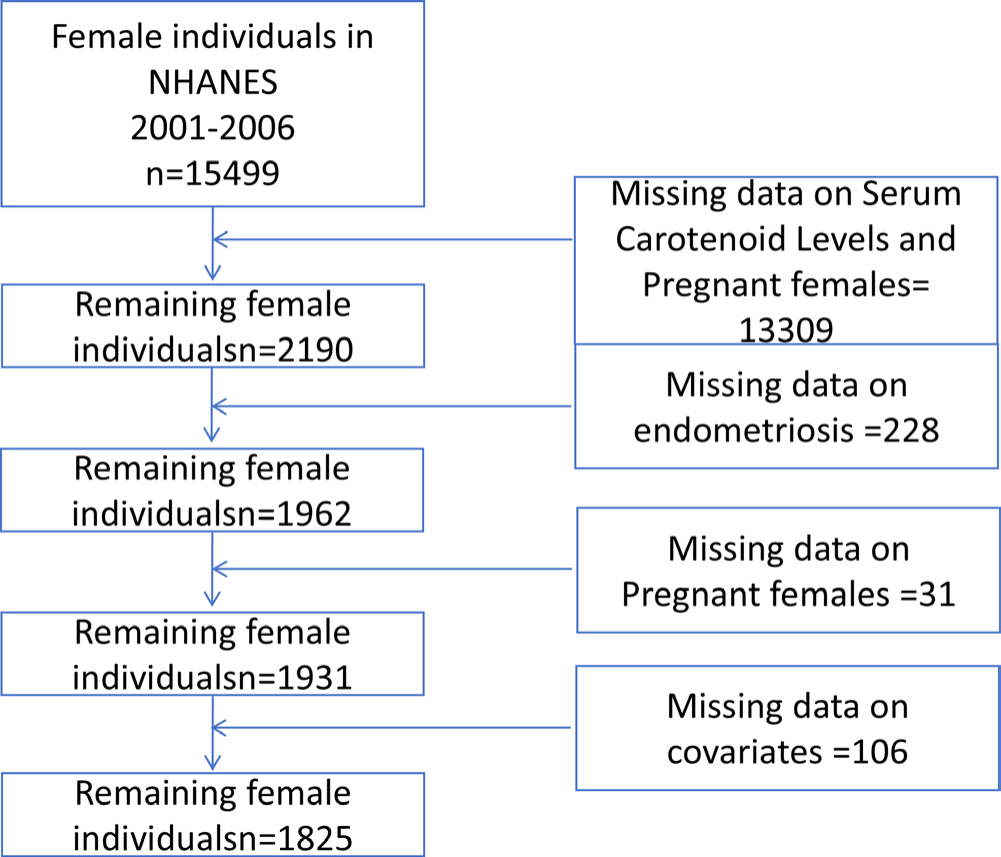
Flow chart of literature screening.

This study is a cross-sectional analysis of NHANES data from 2001 to 2006. Female participants aged 20 to 49 years with available data on self-reported endometriosis diagnosis and serum carotenoid levels were included. Participants who were pregnant or had missing data on key covariates were excluded. To account for NHANES complex, multistage probability sampling design, survey weights were applied to ensure nationally representative estimates (Fig. 1). The NHANES survey protocol was approved by the National Center for Health Statistics Research Ethics Review Board. All participants provided written informed consent. For this secondary analysis of publicly available, de-identified data, no additional institutional review board approval was required.

### 2.2. Exposure assessment

Serum levels of α-carotene, β-carotene, β-cryptoxanthin, lutein/zeaxanthin, lycopene, and total carotenoids were measured using high-performance liquid chromatography following NHANES standardized laboratory protocols. Quality control procedures included internal calibration standards and external quality assurance programs. Carotenoid levels were analyzed as continuous variables and categorized into quintiles.

### 2.3. Outcome assessment

Endometriosis diagnosis was determined based on self-reported physician diagnosis during NHANES interviews. Participants who answered “yes” to the question “Has a doctor ever told you that you have endometriosis?” were classified as having endometriosis.

### 2.4. Covariates

Potential confounders were selected based on prior literature and biological plausibility, including – Demographic factors: age, race/ethnicity, education level, and poverty income ratio. Lifestyle factors: BMI and smoking status. Reproductive factors: age at menarche and number of pregnancies.

### 2.5. Statistical analysis

Baseline characteristics were compared between participants with and without endometriosis. Continuous variables were assessed for normality and reported as means (standard deviations) or medians (interquartile ranges). Differences were tested using *t* tests or Wilcoxon rank-sum tests, as appropriate. Categorical variables were presented as frequencies (percentages) and compared using chi-square or Fisher’s exact tests.

Survey-weighted logistic regression models were used to assess the association between serum carotenoid levels (quintiles) and endometriosis prevalence, adjusting for NHANES’s complex sampling design. Three models were constructed – Model 1: Unadjusted. Model 2: Adjusted for age, race/ethnicity, education level, and poverty income ratio. Model 3: Further adjusted for BMI, smoking status, age at menarche, and number of pregnancies.

### 2.6. Subgroup and sensitivity analyses

Subgroup analyses were conducted for BMI, smoking status, and age at menarche to examine potential effect modification. Interaction terms were tested using likelihood ratio tests, and statistical significance for interactions was set at *P* < .10.

Sensitivity analyses included: Excluding participants with extreme carotenoid levels (above the 99th percentile) to assess the impact of outliers. Alternative categorization of carotenoid levels (tertiles instead of quintiles). Comparing survey-weighted and unweighted models to evaluate the robustness of the findings.

All analyses were performed using R software (version 4.2.0), incorporating NHANES survey weights to produce nationally representative estimates. A 2-sided *P*-value < .05 was considered statistically significant.

## 3. Results

### 3.1. Baseline characteristics

A total of 1825 participants were included in the final analysis, representing a weighted population of 3,22,24,214 individuals. Among them, 99 (5.4%) were diagnosed with endometriosis. As shown in Table 1, participants with endometriosis were more likely to be non-Hispanic White (66.7% vs 42.9%, *P* < .001) and have a higher education level (64.6% vs 51.7%, *P* = .012). They also had a higher smoking rate (50.5% vs 39.9%, *P* = .036). No significant differences were observed for BMI, age at menarche, or the number of pregnancies (all *P* > .05).

**Table 1:** The baseline characteristics according to endometriosis groups.

Characteristic	Overall, N = 1825	Non-endometriosis, N = 1726	Endometriosis, N = 99	*P*-value
α-carotene				.427
Median (IQR)	2.8 (1.5, 5.3)	2.8 (1.5, 5.3)	2.5 (1.5, 4.8)	
β-carotene				.280
Median (IQR)	12 (7, 21)	12 (7, 22)	11 (6, 20)	
β-cryptoxanthin				<.001
Median (IQR)	8 (5, 13)	8 (5, 13)	6 (4, 10)	
Lycopene				.536
Median (IQR)	22 (16, 29)	22 (16, 29)	21 (15, 29)	
Lutein and zeaxanthin				.008
Median (IQR)	13 (10, 19)	14 (10, 19)	12 (8, 17)	
Total serum carotenoids				.065
Median (IQR)	63 (47, 85)	63 (47, 85)	58 (42, 78)	
Race				<.001
Mexican American	442 (24.2%)	433 (25.1%)	9 (9.1%)	
Other Hispanic	74 (4.1%)	70 (4.1%)	4 (4.0%)	
Non-Hispanic White	806 (44.2%)	740 (42.9%)	66 (66.7%)	
Non-Hispanic Black	429 (23.5%)	412 (23.9%)	17 (17.2%)	
Others	74 (4.1%)	71 (4.1%)	3 (3.0%)	
Age				.041
<32	545 (29.9%)	526 (30.5%)	19 (19.2%)	
32–41	586 (32.1%)	546 (31.6%)	40 (40.4%)	
≥41	694 (38.0%)	654 (37.9%)	40 (40.4%)	
Education				.012
High school or equivalent	869 (47.6%)	834 (48.3%)	35 (35.4%)	
College or above	956 (52.4%)	892 (51.7%)	64 (64.6%)	
PIR				.368
≤1.3	576 (31.6%)	543 (31.5%)	33 (33.3%)	
1.3–3.5	689 (37.8%)	658 (38.1%)	31 (31.3%)	
>3.5	560 (30.7%)	525 (30.4%)	35 (35.4%)	
BMI				.347
<24	517 (28.3%)	483 (28.0%)	34 (34.3%)	
24–28	444 (24.3%)	420 (24.3%)	24 (24.2%)	
≥28	864 (47.3%)	823 (47.7%)	41 (41.4%)	
Age at menarche				.615
≤13	1362 (74.6%)	1286 (74.5%)	76 (76.8%)	
>13	463 (25.4%)	440 (25.5%)	23 (23.2%)	
Number of pregnancies				.649
≤3	1234 (67.6%)	1165 (67.5%)	69 (69.7%)	
>3	591 (32.4%)	561 (32.5%)	30 (30.3%)	
Smoke				.036
0	1087 (59.6%)	1038 (60.1%)	49 (49.5%)	
1	738 (40.4%)	688 (39.9%)	50 (50.5%)	

n (%).

Wilcoxon rank sum test.

Fisher’s exact test.

Pearson’s chi-squared test.

BMI = body mass index, IQR = interquartile range, PIR = poverty income ratio.

### 3.2. Association between serum carotenoids and endometriosis

Survey-weighted logistic regression analysis (Table 2) indicated that higher serum levels of α-carotene (odds ratio [OR] = 0.94, 95% confidence interval [CI]: 0.89–0.99, *P* = .048), β-carotene (OR = 0.98, 95% CI: 0.96–1.00, *P* = .038), lycopene (OR = 0.95, 95% CI: 0.91–0.99, *P* = .021), and total carotenoids (OR = 0.99, 95% CI: 0.98–1.00, *P* = .044) were significantly associated with a lower prevalence of endometriosis. However, β-cryptoxanthin (OR = 0.94, 95% CI: 0.88–1.01, *P* = .12) and lutein/zeaxanthin (OR = 1.00, 95% CI: 0.97–1.03, *P* = .92) were not significantly associated with endometriosis risk.

**Table 2:** Association between serum carotenoids and endometriosis.

Characteristic	Model A	Model B	Model C
	OR (95% CI) *P*-value	OR (95% CI) *P*-value	OR (95% CI) *P*-value
α-carotene	0.95 (0.91, 0.99) .024*	0.94 (0.89, 0.99) .017*	0.94 (0.89, 0.99) .025*
β-carotene	0.98 (0.97, 1.00) .032*	0.98 (0.96, 1.00) .029*	0.98 (0.96, 1.00) .038*
β-cryptoxanthin	0.93 (0.88, 0.99) .027*	0.94 (0.88, 1.00) .059	0.94 (0.88, 1.01) .073
Lycopene	0.95 (0.91, 0.99) .011*	0.95 (0.91, 0.99) .011*	0.95 (0.91, 0.99) .014*
Lutein and zeaxanthin	1.00 (0.97, 1.02) .764	1.00 (0.97, 1.03) .769	1.00 (0.97, 1.03) .797
Total serum carotenoids	0.99 (0.98, 1.00) .013*	0.99 (0.98, 1.00) .015*	0.99 (0.98, 1.00) .021*

Cl = confidence interval, OR = odds ratio. * *P*-values are considered statistically significant, using the common threshold of *P* < .05.

### 3.3. Subgroup and sensitivity analyses

**Figure 2.**
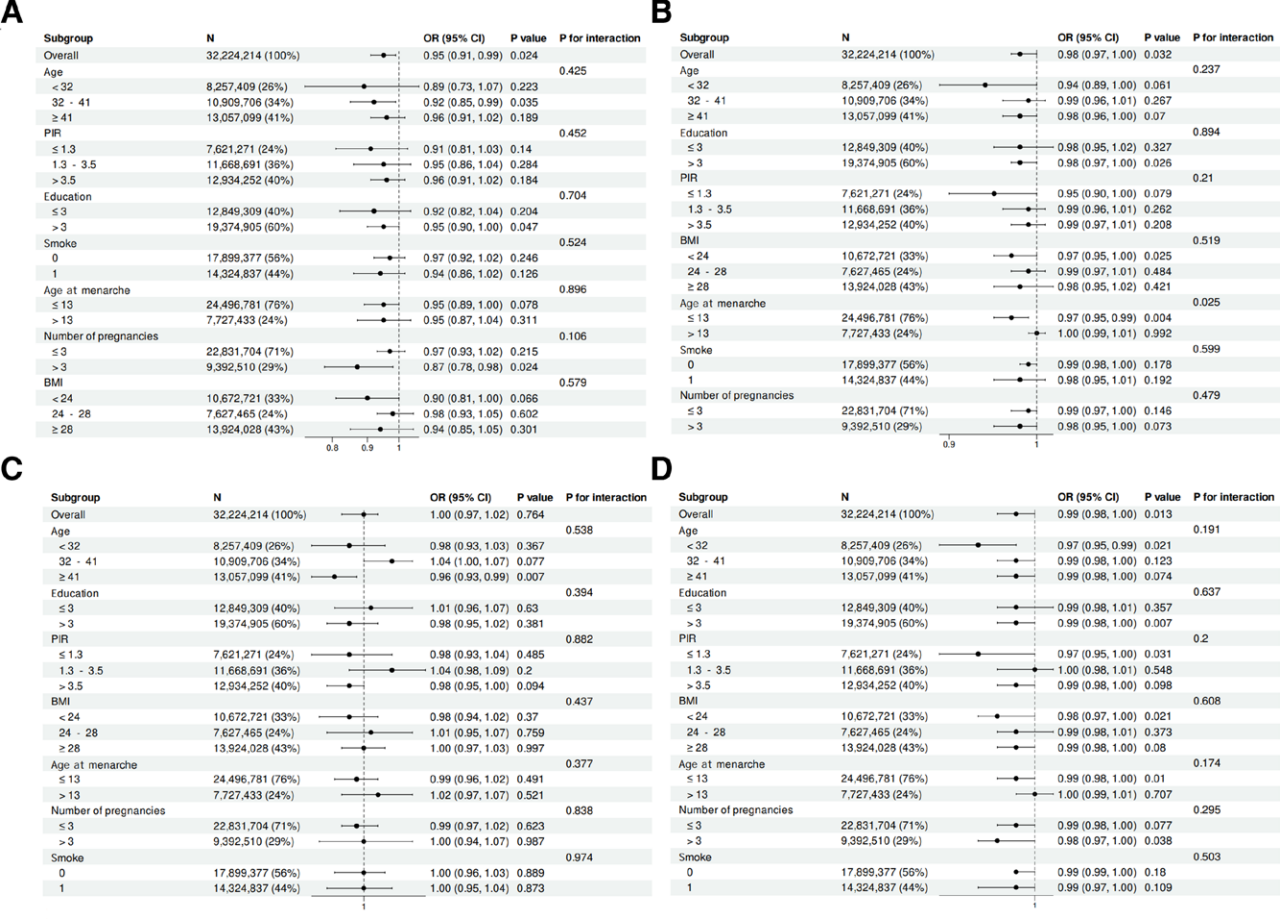
Subgroup and sensitivity analyses. (A) Subgroup analysis of α-carotene; (B) subgroup analysis of β-carotene; (C) subgroup analysis of lycopene; (D) subgroup analysis of total carotenoids.

As shown in Figure 2, subgroup analysis revealed a significant interaction between β-carotene levels and age at menarche in relation to endometriosis prevalence (*P*-interaction < .05). No significant interactions were observed for BMI or smoking status (all *P* > .05). Sensitivity analyses confirmed the robustness of the findings, as excluding participants with extreme carotenoid levels (>99th percentile) or using alternative categorization methods (tertiles instead of quintiles) did not substantially alter the results.

## 4. Discussion

This study provides epidemiological evidence that higher serum levels of α-carotene, β-carotene, lycopene, and total carotenoids are significantly associated with a lower prevalence of endometriosis, whereas β-cryptoxanthin and lutein/zeaxanthin do not exhibit significant associations. These findings suggest that specific carotenoids may contribute to endometriosis prevention, possibly through their antioxidative and immunomodulatory effects.

Although limited studies have specifically investigated carotenoids and endometriosis, existing research supports the potential protective role of antioxidant-rich diets in gynecological conditions. A large prospective cohort study reported that higher intake of fruits and vegetables – major dietary sources of carotenoids – was associated with a reduced risk of endometriosis.^[12,13]^ Furthermore, β-carotene intake has been linked to decreased inflammatory markers, suggesting a potential immunomodulatory role in suppressing endometriotic lesion formation.^[14–16]^ These findings align with the current study, reinforcing the hypothesis that carotenoids may contribute to reducing the burden of endometriosis.

Oxidative stress plays a crucial role in the pathophysiology of endometriosis, promoting chronic inflammation, immune dysregulation, and cellular damage. Elevated ROS in the peritoneal fluid of affected individuals have been associated with increased expression of pro-inflammatory cytokines, such as interleukin-6 and tumor necrosis factor-alpha, which exacerbate disease progression.^[17–19]^ Carotenoids, particularly α-carotene, β-carotene, and lycopene, are known to neutralize ROS, thereby mitigating oxidative stress-induced cellular injury. Additionally, carotenoids have been shown to downregulate NF-κB and upregulate Nrf2 pathways, which regulate inflammation and oxidative stress responses.^[20,21]^ These mechanisms may explain the observed inverse associations between specific carotenoids and endometriosis prevalence.

Interestingly, β-cryptoxanthin and lutein/zeaxanthin did not show significant associations with endometriosis in this study. One possible explanation is the differences in bioavailability and metabolic pathways among carotenoids. Unlike β-carotene and lycopene, lutein/zeaxanthin are primarily localized in ocular tissues, with limited systemic antioxidant activity relevant to reproductive health.^[9,22,23]^ Furthermore, genetic polymorphisms affecting carotenoid metabolism may lead to interindividual differences in their biological effects, potentially contributing to the null associations observed.

This study has several strengths. The use of NHANES data ensures a nationally representative sample, enhancing the generalizability of the findings. The application of survey-weighted logistic regression models accounts for NHANES’s complex sampling design, providing robust statistical estimates.

However, several limitations must be acknowledged. First, the cross-sectional design precludes causal inferences, and the observed associations should be interpreted cautiously. Second, endometriosis diagnosis was self-reported, which may introduce recall bias and misclassification errors. Third, serum carotenoid levels were measured at a single time point, which may not accurately reflect long-term dietary intake or fluctuations in carotenoid status. Additionally, this study did not evaluate potential dose-response relationships, limiting the ability to determine whether increasing carotenoid intake beyond a certain threshold provides additional benefits. Finally, despite adjusting for multiple confounders, residual confounding from unmeasured factors such as dietary patterns, physical activity, and environmental exposures cannot be ruled out.

## 5. Conclusion

In summary, this study suggests that higher serum levels of α-carotene, β-carotene, lycopene, and total carotenoids may be associated with a lower prevalence of endometriosis, supporting the potential role of dietary carotenoids in mitigating endometriosis risk. Future prospective studies and mechanistic research are warranted to confirm these associations and elucidate the biological pathways underlying the protective effects of carotenoids on endometriosis development.

## Author contributions

**Conceptualization:** Zhuoxia Chen, Tingting Zhang.

**Data curation:** Zhuoxia Chen, Tingting Zhang, Tiantian Jin.

**Formal analysis:** Tiantian Jin.

**Writing – original draft:** Zhuoxia Chen, Tingting Zhang, Tiantian Jin.

**Writing – review & editing:** Zhuoxia Chen, Tingting Zhang, Tiantian Jin.
